# Cholera trends in the WHO African region and temporal co-occurrence with El Niño, tropical cyclones, floods and droughts: 2022–2023

**DOI:** 10.1136/bmjph-2025-004479

**Published:** 2026-08-12

**Authors:** Otim Ramadan, Adebola Tolulope Olayinka, Felix Sanni, Susan Nyawade, Olaolu Moses Aderinola, Olushayo Oluseun Olu, Rashidatu Kamara, Tendai Makamure, John Masina, Fred Dratibi, Deborah Barasa, Charles Okot, Ishata Nannie Conteh, Mory Keita, Joseph Okeibunor, Dick Damas Chamla, Fiona Braka, Abdou Salam Gueye, Chikwe Ihekweazu

**Affiliations:** World Health Organization, Regional Office for Africa, Brazzaville, Republic of Congo

**Keywords:** Epidemics, Disease Transmission, Infectious, Water Pollution, Public Health

## Abstract

**Introduction:**

Cholera is an indicator of inequity and lack of social development and it majorly affects poor and vulnerable people. Cholera outbreaks linked to seasonality continue to occur unabated in Africa, necessitating better understanding of the disease trends. This study describes the trend of cholera outbreaks in the AFRO region between January 2022 and December 2023 and examines the temporal co-occurrence with major climatic events at the period.

**Method:**

We undertook a descriptive ecological trend analysis of secondary surveillance data on cholera and overlaid outbreak trends with the timing of major climate events from 1 January 2022 to 31 December 2023 from affected countries in the AFRO region. Negative binomial regression with country fixed effects and seasonal harmonics was used to quantify the association between climate event exposure and weekly cholera incidence.

**Results:**

Cumulative cases and deaths reported in 2022 were 90 227 and 2296, with case fatality ratio (CFR) of 2.55% across nine countries. In 2023, a total of 197 132 cases and 2830 deaths (CFR 1.4%) were reported from 17 countries, with the number of affected countries nearly doubling. Population-adjusted attack rates increased from 13.5 per 100 000 in 2022 to 21.9 per 100 000 in 2023. Climatic events ranging from droughts, excessive rainfall, floods and tropical cyclones occurred at different times across the 2-year study period. Negative binomial regression showed that cyclone (incidence rate ratio (IRR) 3.88, 95% CI 2.57 to 5.85), flood (IRR 1.93, 95% CI 1.46 to 2.54) and drought exposures (IRR 3.89, 95% CI 2.93 to 5.16) were each independently associated with higher cholera incidence (all p<0.001).

**Conclusion:**

The seasonality of cholera outbreaks and climatic events continue to be a challenge for countries to deal with. Between 2022 and 2023 in the African region, extreme weather events overlapped with waves of cholera epidemics. It is important to integrate preparedness for cholera and control into contingency planning for climatic events.

**WHAT IS ALREADY KNOWN ON THIS TOPIC**

- Cholera, a disease associated with poor social development, poor access to water, sanitation, hygiene infrastructure and poverty, causes seasonal epidemics in Africa.

**WHAT THIS STUDY ADDS**

- Between 2022 and 2023 in the WHO African region, subregions that experienced major climate events, such as droughts, severe rains, floods, tropical storms and/or cyclones showed a temporal overlap with increases in cholera cases.

**HOW THIS STUDY MIGHT AFFECT RESEARCH, PRACTICE OR POLICY**

- Cholera preparedness and readiness should incorporate early warning systems for adverse climatic conditions into planning and adequately mitigate the effects and impact of concurrent cholera outbreaks and climatic health events.

## Introduction

Cholera is an ancient disease that remains a global public health threat with an annual estimate of 4 million cholera cases and over 140 000 deaths.^1^ It was first described in fifth to fourth-century BCE and second to third-century CE by Hippocrates and Galen, respectively. The symptoms were described by a historian, Gasspar Corean in 1543 in his account of the ‘moryxy’ epidemic in India.^2^ Cholera is an acute watery diarrhoeal infection caused by ingestion of food or water contaminated with the bacterium *Vibrio cholerae,* with the severe form of the disease leading to death within hours if left untreated.^1^ The disease serves as an indicator of inequity and lack of social development as it majorly affects and kills the poor and most vulnerable groups of people.^3^ From the first cholera pandemic of 1817 which originated from India, the world has experienced six subsequent pandemics and the current pandemic (the seventh and longest recorded) started in 1961 from South Asia and reached Africa in 1971.^4^ In Africa alone, 40–80 million people live in identified cholera hotspots with recurrent outbreaks linked to seasonality, and this has remained a major public health problem on the continent.^5^ Studies have shown correlation between seasonality, climate changes and incidence and transmission of cholera, especially in endemic regions as these lead to changes in environmental conditions and eventually affect the quantity and quality of safe and available drinking water to the majority of the people.^6–8^

On 4 October 2017, the Global Task Force on Cholera Control (GTFCC) officially launched a strategy named: Ending Cholera—A Global Roadmap to 2030. This was followed by a resolution in 2018 to end cholera, which was adopted at the 71st World Health Assembly,^5^ after which the WHO Regional Office for Africa (WHO AFRO) developed a Regional Implementation framework with key milestones to guide member states to implement the elimination of cholera by 2030 global strategy.^9^ The framework proposes enhancing epidemiological and laboratory surveillance, mapping cholera hotspots, improving access to timely treatment, strengthening partnerships, community engagement, increasing investments in clean water and sanitation for the most vulnerable communities and promoting research.^9^

However, despite the ongoing implementation of the African response framework, cholera outbreaks have continued to occur unabated in the region necessitating better understanding of the epidemiology and trend of the outbreak across seasons and the distribution and pattern of the disease across subregions on the African continent. Countries have been supported to conduct comprehensive assessments and use those to develop National Cholera elimination plans, hotspot mappings have been done and in recent times identification of Priority Areas for Multisectoral Interventions (PAMIs) all as avenues to move towards reduction of the occurrence of outbreaks of cholera and attendant morbidity and mortality with 23 countries in the WHO African region having identified PAMIs.^10^

Cholera outbreaks, however, continue to be reported annually from several countries, especially in sub-Saharan Africa. Previous studies have estimated that 2.9 million cases and 95 000 deaths occur annually,^11^ but the proportion of people who die from cholera is estimated to be about 100 000–120 000 000 of 3–5 million cases probably occurring annually^12^ but unreported. It is also estimated that 1.4 billion of the world’s population is at risk for cholera, with countries in Africa and southern Asia being the countries with the highest incidence rates.^13^

The aim of this study is to describe the cholera outbreak trends in the WHO African region between January 2022 and December 2023, characterise successive waves and assess temporal co-occurrence between cholera seasonality and major climate events across subregions.

## Methodology

The study used a descriptive ecological design based on secondary surveillance data from 1 January 2022 to 31 December 2023, obtained from affected countries in the WHO African region as part of outbreak response. Reported data on suspected cholera cases and deaths were compiled from various authoritative platforms—these are country and regional situation reports, national public health bulletins, publicly available country-specific dashboards, through the Integrated Disease Surveillance and Response and District Health Information Software 2 systems and from national aggregated Excel sheets and national line lists as submitted to WHO AFRO. National aggregated Excel sheets and line lists are collated by national surveillance teams and shared voluntarily by countries with the WHO Cholera Incident Management Support Team surveillance pillar during outbreaks. Countries with at least one cholera case confirmed by positive culture or PCR test were included as consistent with the GTFCC outbreak definition^14^ while data from countries which report to the Eastern Mediterranean WHO regional office, even though situated in Africa, were excluded. The excluded countries are Djibouti, Egypt, Libya, Morocco, Somalia, Sudan and Tunisia. Because reporting systems differed by country and over time, analyses were restricted to aggregated weekly counts and interpreted as regional trend patterns rather than directly comparable incidence measurements across all countries. Climate event timing and affected-country classification were documented using datasets and operational sources, including International Best Track Archive for Climate Stewardship tropical cyclone tracks, National Oceanic and Atmospheric Administration, Oceanic Niño Index (ONI), ECMWF Reanalysis V.5, Climate Hazards Group InfraRed Precipitation with Station data rainfall data and Emergency Events Database (EM-DAT) disaster records.^15–19^ In this manuscript, an epidemic wave is operationally defined as a sustained rise followed by decline in weekly reported cases. No smoothing or moving averages were applied to the epidemic curves; all figures display raw weekly aggregated counts.

Data entry, cleaning and basic descriptive analyses were conducted using Microsoft Excel. Weekly, monthly and yearly outbreak trends and the timing of climate events are presented as tables and charts. Epidemiological curves were generated using R software. Association between climate events and cholera incidence was determined by fitted negative binomial regression modelling. The negative binomial distribution was chosen over Poisson regression to account for overdispersion. The primary model included binary indicators for cyclone exposure (country-weeks during or within 6 weeks after a named tropical cyclone landfall), flood exposure (country-weeks during a major flood event recorded in EM-DAT) and drought exposure (country-weeks during Integrated Food Security Phase Classification—phase III or higher=Crisis, Emergency, or Famine). Phase III or higher drought conditions as classified by Famine Early Warning Systems Network, with the ONI as a continuous covariate. Country fixed effects controlled for time-invariant between-country differences and Fourier harmonic terms (annual and semi-annual sine and cosine pairs) captured seasonal patterns in cholera transmission. Log-transformed national population served as an offset to model incidence rates. Results are presented as incidence rate ratios (IRR) with 95% CIs. Cross-correlation analysis between the ONI time series and aggregated weekly case counts were performed at lags from 0 to 26 weeks to explore temporal lead-lag relationships between El Niño-Southern Oscillation (ENSO) state and regional cholera activity (online supplemental table S1). Population-adjusted attack rates were calculated per 100 000 population per year using national population estimates from WHO AFRO surveillance records. Sensitivity analyses were performed using (1) binary El Niño and La Niña indicators in place of continuous ONI and (2) a single combined climate event indicator to assess the robustness of the primary model estimates. All statistical analyses were performed using R and Python (stats models and scipy libraries).

10.1136/bmjph-2025-004479.supp1 Supplementary data

The regression model was specified a priori; covariates were selected on the basis of biological plausibility and data availability rather than by automated stepwise procedures, with cyclone, flood and drought exposures and the ONI as the exposures of interest and country fixed effects, the calendar-year indicator and the Fourier seasonal terms retained as structural adjustments in all models. Multicollinearity among covariates was assessed using variance inflation factors (VIFs). Overdispersion in the weekly counts was confirmed before model fitting (Poisson dispersion statistic far exceeding 1; estimated negative binomial dispersion parameter α ≈ 0.9, p<0.001), justifying the negative binomial specification over Poisson regression, and model fit was summarised using the Akaike information criterion (AIC), the deviance-to-degrees-of-freedom ratio and McFadden’s pseudo-R². Repeated weekly observations within the same country were accounted for through country fixed effects; as a sensitivity analysis, CIs were additionally re-estimated using cluster-robust (sandwich) SEs grouped by country.

Stringent data management protocols, including confidentiality, anonymity and data security, were employed to ensure data protection and integrity.

## Results

In the year 2022, the cumulative cases and deaths of cholera reported to WHO AFRO were 90 227 and 2296, with a case fatality ratio (CFR) of 2.5%, across nine countries (Burundi, Cameroon, Democratic Republic of Congo, Ethiopia, Kenya, Malawi, Mozambique, Nigeria and Zambia). Following this figure, in the next (first) month of the new year, as of 31 January 2023, there were already 13 515 cases and 218 deaths (CFR 1.6%) reported in the region. In year 2023, a total of 197 132 cases and 2830 deaths (CFR 1.4%) were reported from 17 affected countries in the WHO African region. The countries were Burundi, Cameroon, Congo, Democratic Republic of Congo, Eswatini, Ethiopia, Kenya, Malawi, Mozambique, Nigeria, South Africa, South Sudan, Tanzania, Togo, Uganda, Zambia and Zimbabwe.

**Figure 1:**
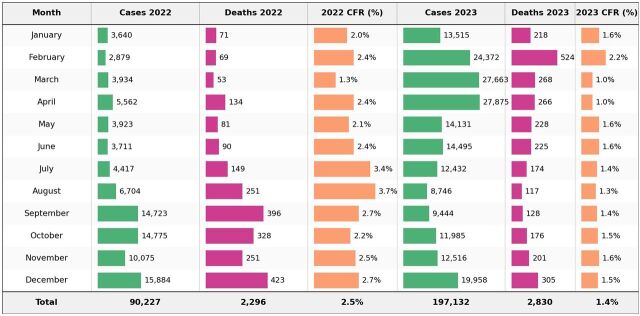
Monthly trends of cholera cases, deaths and case fatality ratio (CFR) within the WHO African region from 1 January 2022 to 31 December 2023.

From January 2022 to 31 December 2023, a cumulative total of 287 359 cases, 5126 deaths and CFR 1.8% (figure 1) were recorded. The number of countries that reported cases in the region almost doubled in 2023 compared with 2022. There was an increase in geographical spread in 2023, which involved countries not endemic for cholera, for example, Eswatini, South Africa and high number of cases from Democratic Republic of Congo, Malawi, Mozambique and Ethiopia, which together contributed 78.0% of cases reported in the region in that year (table 1).

**Table 1:** Distribution of cumulative cholera cases, deaths, case fatality ratios and population-adjusted attack rates from affected countries reporting to the WHO Regional Office for Africa for 2022 and 2023

Country	Cases 2022	Deaths 2022	Case fatality ratio (%) 2022	Attack rates 2022	Cases 2023	Deaths 2023	Case fatality ratio (%) 2023	Attack rates 2023
Democratic Republic of the Congo	18 394	322	1.8	16.3	51 981	439	0.8	46.1
Malawi	27 384	1004	3.7	123.3	31 721	767	2.4	142.8
Mozambique	1352	5	0.4	3.8	39 535	156	0.4	111.0
Ethiopia	893	27	3.0	0.7	30 528	446	1.5	22.5
Nigeria	23 839	597	2.5	10.0	3683	128	3.5	1.6
Cameroon	14 124	275	1.9	0.0	6477	209	3.2	0.0
Zimbabwe	–	–	–	–	14 517	320	2.2	85.6
Kenya	4017	66	1.6	7.0	8354	141	1.7	14.5
Zambia	16	0	0.0	0.1	5212	136	2.6	23.8
South Sudan	–	–	–	–	1471	2	0.1	12.1
Burundi	25	0	0.0	0.2	1345	9	0.7	9.6
Tanzania	–	–	–	–	1128	21	1.9	1.6
South Africa	–	–	–	–	1057	27	2.6	1.6
Uganda	–	–	–	–	84	10	11.9	0.2
Republic of Congo	–	–	–	–	63	0	0.0	1.0
Eswatini	–	–	–	–	2	0	0.0	0.2
Togo	–	–	–	–	1	0	0.0	0.0
Total	90 227	2296	2.5	14.1	197 132	2830	1.4	22.7

Burkina Faso (3), Rwanda (24) and Liberia (367) suspected/unconfirmed cases in 2022.

In the WHO African region by January 2023, new cases increased by almost 30% of the total cases in 2022 and the first four months of the year (January–April) accounted for 42.6% of total cases and 45.3% of total deaths within the year. Top reporting countries for year 2023 were the Democratic Republic of Congo, Mozambique, Malawi and Ethiopia, accounting for 78.0% of total cases reported in 2023 (figure 1, table 1, online supplemental figure S1).

10.1136/bmjph-2025-004479.supp2 Supplementary data

**Figure 2:**
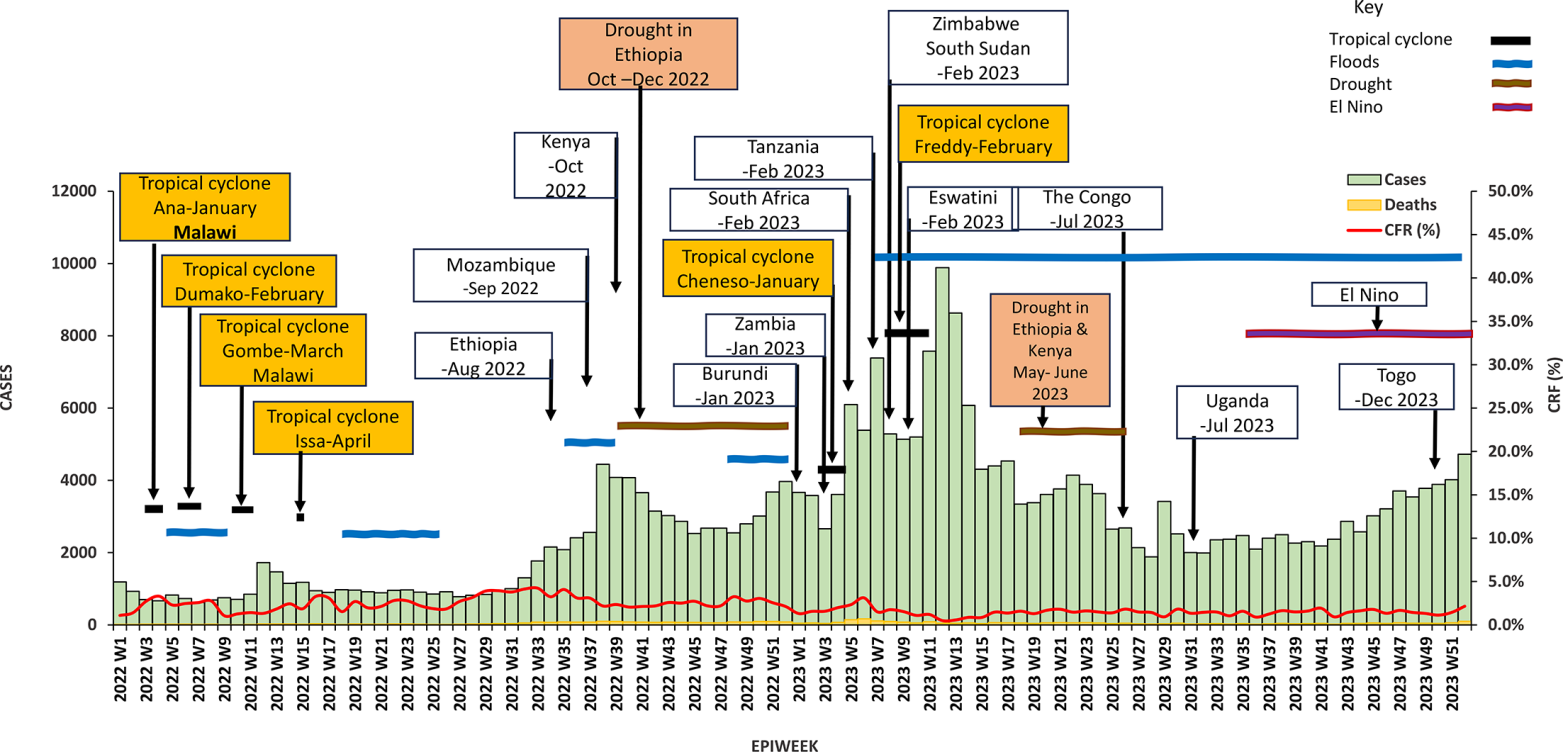
Trendline of cholera cases showing periods of climatic events and affected countries in the African region from 1 January 2022 to 31 December 2023. CFR, case fatality ratio.

Population-adjusted annual cholera attack rates increased from 13.5 per 100 000 in 2022 to 21.9 per 100 000 in 2023 across the 17 reporting countries (table 1). The highest attack rates in 2023 were observed in Malawi (142.8 per 100 000), Mozambique (111.0 per 100 000), Zimbabwe (85.6 per 100 000) and Democratic Republic of Congo (46.1 per 100 000). In the year 2023, three regional epidemic waves were observed from weekly aggregated counts: weeks 5–14 (major peak; maximum 9742 weekly cases), weeks 15–24 (prolonged moderate elevation) and weeks 46–52 (late-year resurgence; maximum 5033 weekly cases). In 2022, the principal wave occurred between weeks 37 and 52, with a peak of 4390 weekly cases. Weekly CFR varied in both years; in 2023, weekly CFR was below the 1.0% target during several weeks, while in 2022 CFR exceeded 4% during part of the year. During 2022–2023, droughts, floods, tropical storms and tropical cyclones affected multiple AFRO countries, with some countries experiencing concurrent or sequential hazards (eg, Ethiopia and Kenya; Malawi and Mozambique). These temporal overlaps are shown in figure 2 and are interpreted as descriptive co-occurrence.

The subregional trend of cholera on the African continent in the past two years has shown significant variability in the number of cases reported across different subregions. The southern subregion experienced three major waves, the first was between September 2022 and April 2023, which started with Malawi being the initially affected country but with Mozambique coming up also. The second wave began from March 2023 and went on till July 2023. The third wave started October 2023, which continued till the end of the year was driven by outbreaks in Zambia, Zimbabwe and Mozambique. Cases in the central subregion started rising from September 2022 and continued to rise without witnessing a decline by the end of year 2023, with Democratic Republic of Congo being the major country affected. For the eastern part of Africa/Greater Horn of Africa the first wave was from October to December 2022 driven primarily by the outbreak in Kenya. This was followed closely by a second wave that started in January 2023 and persisted till the end of the year with major outbreaks in Ethiopia, Kenya and also Tanzania. By June 2023, the outbreak in Kenya had waned and the outbreak for the second half of the year was mainly by Ethiopia and also with contributions from Tanzania. From the West African subregion, Nigeria, the main country reporting had relatively high number of cases by end of 2022 and first quarter of 2023 but from March 2023, experienced low transmission of the outbreak (online supplemental figure S2).

10.1136/bmjph-2025-004479.supp3 Supplementary data

**Figure 3:**
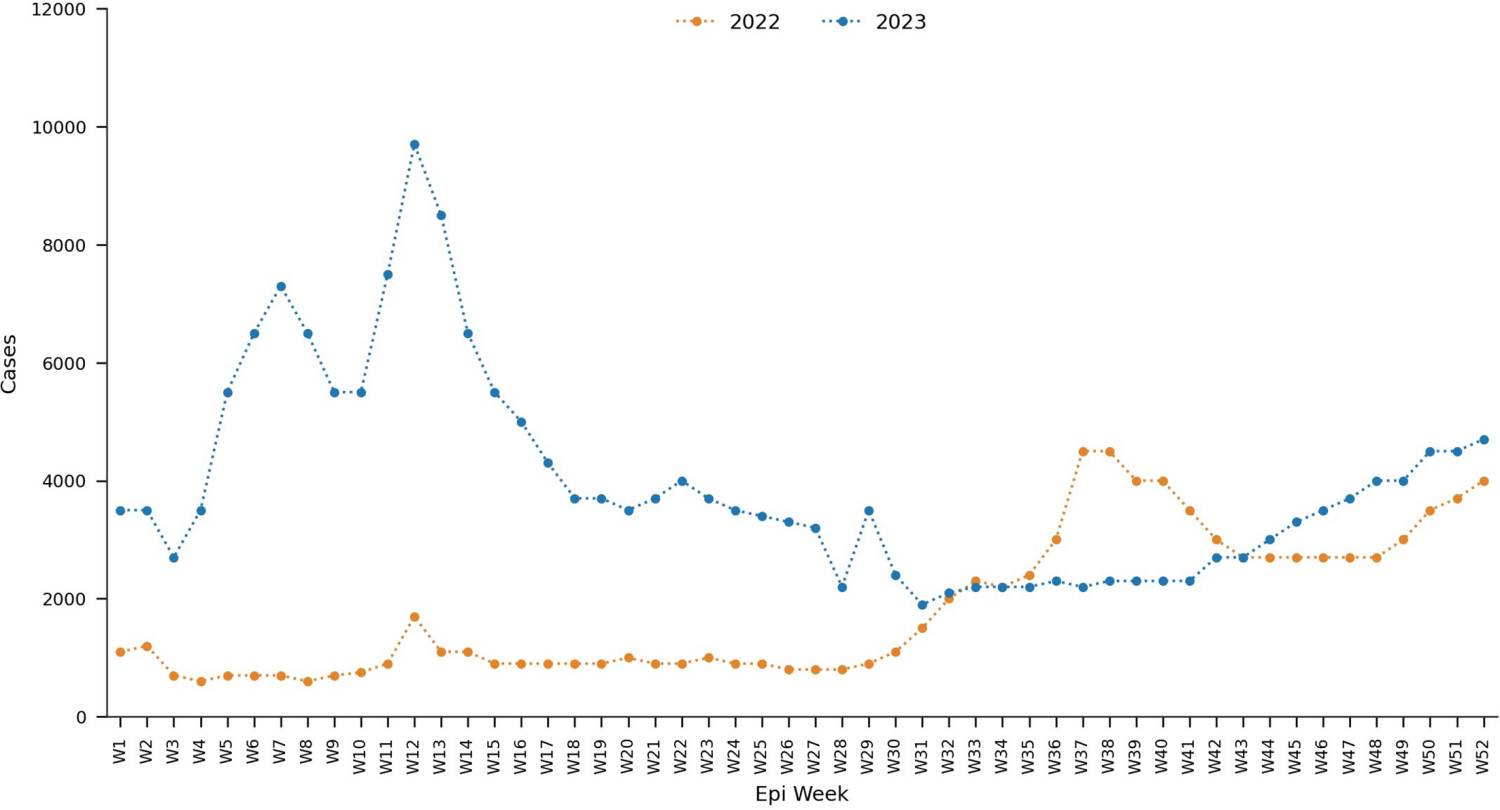
Comparison of weekly epidemic trendlines of cholera cases in the WHO African region for 2022 and 2023.

The weekly reported cholera cases in 2023 surpassed those for the same period in 2022 when compared across each month of the year except between epidemiological weeks 39–43, when the cases in 2022 were higher than the corresponding period in 2023 (figure 3). Negative binomial regression with country fixed effects, seasonal harmonics and population offset showed that tropical cyclone exposure was associated with a 3.9-fold increase in weekly cholera incidence (IRR 3.88, 95% CI 2.57 to 5.85, p<0.001), flood exposure with a 1.9-fold increase (IRR 1.93, 95% CI 1.46 to 2.54, p<0.001) and drought exposure with a 3.9-fold increase (IRR 3.89, 95% CI 2.93 to 5.16, p<0.001). The ONI, as a continuous measure of ENSO state, showed a borderline inverse association with cholera incidence (IRR 0.85, 95% CI 0.73 to 0.98, p=0.030), though this was not robust across alternative model specifications. In a combined model, all climate event exposures were associated with a 3.2-fold increase in cholera incidence (IRR 3.20, 95% CI 2.65 to 3.87, p<0.001).

During cyclone-exposed country-weeks, mean weekly cases per country were 5.3 times higher than in unexposed periods (Mann-Whitney U, p<0.001). Sensitivity analyses using binary El Niño/La Niña indicators and a combined climate event indicator yielded consistent results: neither El Niño nor La Niña was independently significant, while the combined climate event indicator was associated with a 3.2-fold increase in cholera incidence (IRR 3.20, 95% CI 2.65 to 3.87, p<0.001). Table 2 presents the full regression results, and the summary of the cholera burden during versus outside climate events (online supplemental table S2) lists the specific climate events coded in the analysis. The negative binomial model showed adequate fit (AIC 11 380.9; log-likelihood −5664.5; 955 country-weeks; deviance/df=1.81; McFadden pseudo-R² = 0.13) and all VIFs were below 6 (climate-hazard exposures 1.1–1.2), indicating no problematic multicollinearity. When CIs were re-estimated using cluster-robust SEs grouped by 17-country clusters, cyclone exposure remained statistically significant (IRR 3.88, p=0.001), whereas the intervals for flood and drought widened and included the null value, reflecting the small number of country clusters and reinforcing a cautious, associational interpretation of the estimates (online supplemental tables S3–S7).

**Table 2:** Negative binomial regression: association between climate factors and weekly cholera incidence

Variable	Incidence rate ratio	95% CI	P value
Oceanic Niño Index (continuous)	0.851	0.735 to 0.984	0.030
Cyclone exposure	3.878	2.573 to 5.845	<0.001
Flood exposure	1.928	1.461 to 2.544	<0.001
Drought exposure	3.887	2.927 to 5.162	<0.001
Year 2023 (vs 2022)	1.231	0.888 to 1.706	0.212

Cholera burden during versus outside climate events					
Event type	Exposed weeks	Mean cases (exposed)	Mean cases (unexposed)	Ratio	P value
Tropical cyclone	34	1383.6	260.8	5.31	<0.001
Flood	65	485.9	287.2	1.69	<0.001
Drought	94	321.2	298.5	1.08	<0.001
Any climate event	184	576.0	235.1	2.45	<0.001

Model fit: AIC = 11380.9; log-likelihood = –5664.5; N = 955 country-weeks.

Exposed weeks = country-weeks during the specified climate event. P value from Mann-Whitney U test (two-sided).

## Discussion

Cholera is endemic in many countries and constitutes a substantial health burden within the WHO African region. Since 2017, the region has witnessed continuing cholera outbreaks. Between years 2022 and 2023, the region experienced a concerning increase in the incidence of cholera outbreaks with an increase in magnitude—in terms of number of countries affected and the doubling of case numbers across the continent. This study period also coincided with the occurrence of various climatic events—tropical cyclones, storms, floods and droughts in the region. The temporal concurrence with these climatic events is consistent with plausible sensitivity of cholera transmission to climate events. This is similar to past events where excessive rainfalls and floods have contributed to an increase in acute watery diarrhoea and cholera cases.^20^ Also the trend seen in the region was similar to the global picture where globally, more than half of the total cholera cases during the past decade were recorded in 2022 and 2023. A total of 472 697 cholera cases and 2349 deaths were reported from 44 countries to the WHO in 2022, which was more than double the 223 370 cases reported from 35 countries in 2021.^21^ Additionally, cholera outbreaks in 2022 were both more frequent and larger. And in 2023, more than 708 200 cases of cholera/acute watery diarrhoea cases, including over 4300 deaths, were reported from 30 countries worldwide.^22^

In 2022, Malawi had the highest number of reported cases, deaths and CFR in the region. The country had experienced tropical cyclones Ana and Dumako between January and February and tropical cyclones Gombe and Issa in March and April of the same year, respectively.^18^ This period coincided with a rise in the number of cholera cases in the southern subregion and the WHO African region between March and April. In our study, analysis revealed that tropical cyclones and droughts were each independently associated with about a fourfold increase in weekly cholera incidence, while flooding was associated with a nearly twofold increase. Understanding also that cyclones and floods damage water and sanitation infrastructure with contamination, populations get displaced into overcrowded temporary shelters with no WASH structures, while droughts concentrate water supplies and reduce the dilution capacity of surface water bodies. This is in line with a previous study, which described the significant impact of climatic events on cholera incidence, especially excessive rainfall and a rise in sea surface temperatures.^23^ There are additional studies that also support that cholera episodes are connected with factors such as environmental variables like significant rainfall and floods with droughts. These events have been associated with the distribution of bacterial diarrhoeal diseases in particular.^24 25^

The first major wave experienced in the period under study occurred between the months of August and October 2022. Countries contributing most cases at this time were Ethiopia, Nigeria, Kenya, Democratic Republic of Congo and Mozambique. In the West African subregion (Nigeria), this period coincided with the rainy season where certain parts of the country experienced flooding. Meanwhile, Ethiopia and the greater Horn of Africa were going through a drought period. This increase in cases as demonstrated by the epidemic wave agrees with previous studies that have demonstrated the effect of excessive rainfall with a resultant increase in cholera transmission, especially where open defaecation is practised.^26^ These flood situations, especially those with severe and increasing rainfalls, end up mobilising enteric pathogens within the environment. Most of these pathogens are usually generated by people, for example, in septic tanks, farmyards (animal faeces) and human faeces where there are no standard latrines. This microbiologically rich material is transmitted into open and shallow wells, rivers, coastal waters and groundwater that are ultimately used for drinking, cooking and other domestic purposes.^27^

This increase in transmission of bacterial pathogens, for example, *V. cholerae,* due to rainfall is not limited to Africa or developing countries alone. In the USA and Canada, waterborne disease outbreaks were found to often be preceded by extreme rainfall events.^28 29^ In the Western Africa subregion during the rainy period, the season seems to be a contributor to the proliferation of cholera and related waterborne illnesses in the region, underscoring the pressing need to understand and address the complex relationship between environmental factors and public health.^30^

The second major wave occurred between January and July 2023. The period started with tropical cyclone Cheneso in January that hit Madagascar, followed by tropical cyclone Freddy in February. These two cyclones affected the southern Africa subregion, with Freddy making landfalls in Mozambique and Malawi, which had existing cholera outbreaks. The severe devastation that followed the event included damage to the WASH infrastructure that further compounded the fragile WASH system in affected communities. Malawi and Mozambique recorded an exponential increase in cases around this period.^31^ This should be expected as it is known that severe weather events apart from causing new cholera outbreaks can also worsen existing ones by the overwhelming of sanitation systems already under strain. This resulted in a rise in cholera cases in the subregion with a marked increase in the number of cases and involvement of more countries. Countries that reported cholera outbreaks between January and February 2023 included Zambia, Burundi, South Africa, Zimbabwe and Eswatini. The African continent has continued to deal with rising trends in cholera outbreaks in recent years, which gets exacerbated by the region’s vulnerability to climate-driven events.^32^

Floods were experienced across the region from July till the end of the year, with Republic of the Congo reporting cholera outbreak during a flood experience mid-year, and this was followed by the El- Niño phenomenon that began from September 2023 till the end of the year with droughts occurring in the southern and eastern subregions of Africa. The El Niño phenomenon caused droughts in Zambia and Zimbabwe and an increase in rainfall levels, causing floods and landslides in some communities (Kenya and Tanzania). Even though there was some decline in cholera cases between July and September, cases started climbing again in the region from late September till the end of the year. Excessive rainfalls and floods have been known to be strongly associated with cholera outbreaks, and past studies had strongly related cholera outbreak events in some African countries to SST spikes due to ENSO events with heavy rainfall and floodings; during drought-induced El Niño events, the risk of cholera remains high.^33^ A study in Wales described a dose–response relationship between drought-related water restrictions and rates of childhood diarrhoea and vomiting.^34^ Other studies have also described the concentration of enteric pathogens in available water bodies during drought periods and the broad distribution of these pathogens with the first rainfall thereafter. Similarly, an outbreak of *Escherichia coli* reported in Eswatini followed the first rainfall after 3 months of drought.^30 35^ An important point in the monthly rainfall trend analysis is that in most months, except January, July, September and October, there was an increase in rainfall in every subregion at varying scales.^36^ The northern, eastern and southern regions also showed a decrease in rainfall during January, July, September and October, respectively which was as expected by usual seasonal changes,

In this study, countries with high case burdens experienced one or more of these climatic events. Democratic Republic of Congo, even though it had other ongoing events such as conflicts with significant population movement, had recorded excessive rainfall and floods and contributed the highest proportion of cases; a similar pattern can be seen in Ethiopia, and the outbreaks in the southern Africa subregion were all related to climate events.

We observed that the weekly CFR in 2023 was better than in 2022. This might have been due to the focus given to cholera preparedness and response in the region following the 2021–2022 outbreaks. In January 2023, WHO AFRO set up a regional cholera IMST and country-level IMSTs that supported member states to coordinate response to the outbreak. Studies have shown that appropriate and early response interventions delivered through a systematic approach as offered through the IMST in recent decades produces dramatic reduction in the size of cholera outbreaks and lead to better outcomes.^37^

This study had some limitations. We had no access to the line list for several countries, and the data received from countries were often incomplete. So, our analyses were restricted to aggregated weekly counts, precluding epidemiological description of age, sex or other individual-level variables. Metadata, such as the proportion of reporting weeks with non-missing data or the proportion of laboratory-confirmed cases among those tested, were not available across all countries, limiting our ability to quantify reporting bias. Second, we are aware that the epidemic waves of cholera experienced in the African region within the study period may not be attributable to climate issues alone; other factors such as water, sanitation and hygiene coverage, functionality of health systems or effects of humanitarian crises with population displacements on the incidence of cholera cases in affected areas were not assessed. We recognise that many factors drive cholera outbreaks and these are diverse: socio-behavioural, poor social development, poor WASH systems, environmental, weak health systems, availability of counter measures, humanitarian crises and population movement,^38–41^ which are prevalent in our region. Third, population-adjusted incidence rates calculated using national denominators do not reflect the focal or subnational concentration of cholera outbreaks and need to be interpreted with caution. Fourth, climate exposures were coded as binary indicators at the national level, which does not capture within-country exposure gradients or dose–response relationships.

Despite these limitations, however, this study has several strengths. First, it provides an overview of the cholera outbreak trend in the WHO African region and the four subregions in the years 2022 and 2023. It also shows the trend pattern during the various major climatic events that occurred in the region within this period. The majority of studies on cholera are either limited to specific countries or attempts to describe the epidemiology or specific interventions for control. This is an ecological analysis based on nationally aggregated data; the reported IRRs therefore quantify temporal associations between climate-hazard exposure and cholera incidence and should not be interpreted as causal effects. Non-climatic drivers—WASH coverage, health-system capacity, conflict and population displacement—were not measured; therefore, residual confounding cannot be excluded. This is a study that describes the trend of a concurrent cholera outbreak, with several climatic events occurring together within the African continent and provides an opportunity for further and more robust study on the topic. This study may have scratched the surface for more rigorous research on climatic events and cholera persistence on the continent.

## Conclusion

In the African region, cholera outbreaks continue to occur in seasonal and climate vulnerable contexts. We observed that epidemic waves of cholera overlapped temporally with extreme weather events, such as droughts, severe rains, floods, tropical storms and/or cyclones, and negative binomial regression showed that each of these hazard types was independently associated with increased cholera incidence after adjusting for country-level differences and seasonality. These events by themselves may not have been the sole cause of the outbreak but may have amplified existing outbreaks and exacerbated existing vulnerabilities such as poor water sanitation and hygiene systems and other factors—environmental, fragile healthcare systems, socio-behavioural and significant population movements due to humanitarian events. Country preparedness plans should therefore strengthen core cholera control capacities, strengthen weak systems and improve early warning signals that should include weather predictions to foster adequate and appropriate health security planning. Emergency preparedness and response to cholera should include climate informed warning to support timely risk mitigation.

## Data Availability

Data are available upon reasonable request. The epidemiologic data that support the findings of this study are not publicly available but on reasonable request can be obtained from the HIS Unit of WHO AFRO and with the permission of the relevant Member States.
