# Supplementary material for: Cholera trends in the WHO African region and temporal co-occurrence with El Niño, tropical cyclones, floods and droughts: 2022–2023
Source: BMJ Public Health. 2026 Aug 12;4(Suppl 1):e004479. doi: 10.1136/bmjph-2025-004479 (PMC13479493; doi:10.1136/bmjph-2025-004479)
Supplement: Supplementary file 1 [file bmjph-4-Suppl_1-s001.pdf]

Supplementary Tables

Table S1. Cross-Correlation Between ONI and Weekly Cholera Cases

Lag (weeks)	R	p-value	Direction
**-26**	0.787	<0.001	Cases lead ONI
**0**	0.274	0.005	Simultaneous
**4**	0.203	0.043	ONI leads cases
**8**	0.131	0.204	ONI leads cases
**12**	0.061	0.562	ONI leads cases
**16**	-0.008	0.942	ONI leads cases
**20**	-0.081	0.462	ONI leads cases
**24**	-0.130	0.252	ONI leads cases

Peak correlation: r = 0.787 at lag -26 weeks (p = 0.0000).

Pearson cross-correlation between NOAA ONI and total weekly cholera case counts across all 17 countries, at various time lags. Positive lag = ONI leads cholera cases.

Table S2. Climate Events coded in the Analysis, 2022 - 2023

Event	Type	Period	Countries	Source
**TC Ana**	Cyclone	2022, weeks 3-4	Mozambique, Malawi	IBTrACS; OCHA Situation Reports
**TC Gombe**	Cyclone	2022, weeks 10-11	Mozambique, Malawi	IBTrACS; OCHA Flash Update
**TC Freddy**	Cyclone	2023, weeks 6-11	Mozambique, Malawi	IBTrACS; WMO longest-lived TC record; OCHA
**Nigeria floods**	Flood	2022, weeks 35-48	Nigeria	EM-DAT; OCHA Nigeria Flood Response
**Cameroon floods**	Flood	2022, weeks 36-42	Cameroon	EM-DAT; OCHA Cameroon Flash Update
**Democratic Republic of the Congo floods**	Flood	2022, weeks 1-8	Democratic Republic of the Congo	EM-DAT; eastern DRC January flooding
**Democratic Republic of the Congo floods**	Flood	2022, weeks 18-26	Democratic Republic of the Congo	EM-DAT; DRC May-June flooding
**Ethiopia floods**	Flood	2022, weeks 14-35	Ethiopia	EM-DAT; OCHA Ethiopia Flash Update
**Tanzania floods**	Flood	2023, weeks 14-22	Tanzania	EM-DAT; OCHA Tanzania situation
**Democratic Republic of the Congo floods**	Flood	2023, weeks 18-22	Democratic Republic of the Congo	EM-DAT; Kalehe catastrophe
**Kenya floods**	Flood	2023, weeks 12-22	Kenya	EM-DAT; OCHA Kenya Flash Update
**Horn/Southern Africa drought**	Drought	2022, weeks 1-52	Ethiopia	FEWS NET; IPC Phase 3-4; OCHA Horn of Africa Drought Response
**Horn/Southern Africa drought**	Drought	2023, weeks 1-18	Ethiopia	FEWS NET; drought broke with Gu rains Mar-May 2023

**Horn/Southern Africa drought**	Drought	2022, weeks 1-52	Kenya	FEWS NET; IPC Phase 3-4; NDMA Kenya drought bulletins
**Horn/Southern Africa drought**	Drought	2023, weeks 1-18	Kenya	FEWS NET; drought broke with Gu rains 2023
**Horn/Southern Africa drought**	Drought	2023, weeks 40-52	Zambia	FEWS NET; ZMD drought advisories; El Nino onset
**Horn/Southern Africa drought**	Drought	2023, weeks 40-52	Zimbabwe	FEWS NET; ZimVAC assessment; El Nino onset

All events are from publicly available, peer-reviewed disaster databases. Cyclone data from IBTrACS; flood data from EM-DAT; drought data from FEWS NET/IPC.

Table S3. Negative Binomial Regression: Association Between Climate Factors and Weekly Cholera Cases

Variable	IRR	95% CI	p-value	Interpretation
**ONI (continuous)**	0.851	0.735 - 0.984	0.030	Per unit increase in ONI (toward El Nino)
**Cyclone**	3.878	2.573 - 5.845	<0.001	Cyclone-exposed vs unexposed country-weeks
**Flood**	1.928	1.461 - 2.544	<0.001	Flood-exposed vs unexposed country-weeks
**Drought**	3.887	2.927 - 5.162	<0.001	Drought-exposed vs unexposed country-weeks
**Year 2023 (vs 2022)**	1.231	0.888 - 1.706	0.212	2023 vs 2022

Model fit: AIC = 11380.9; Log-likelihood = -5664.5; N = 955 country-weeks.

IRR = incidence rate ratio; CI = confidence interval.

Table S4. Goodness-of-fit statistics (primary negative binomial model)

Statistic	Value
Observations (country-weeks)	955
Residual degrees of freedom	929
Log-likelihood	-5664.5
AIC	11380.9
Deviance / df	1.81
Pearson chi-square / df	1.73
McFadden pseudo-R^2	0.128

A deviance/df ratio near 1 and a low AIC indicate adequate fit after accounting for overdispersion via the negative binomial specification.

Table S5. Multicollinearity: variance inflation factors (VIF)

Covariate	VIF
ONI (continuous)	5.74
Cyclone exposure	1.14
Flood exposure	1.09
Drought exposure	1.10
Calendar year (2023 vs 2022)	4.70
sin (annual)	2.36
cos (annual)	1.09
sin (semi-annual)	1.27
cos (semi-annual)	1.07

Table S6. Handling of repeated observations: cluster-robust standard errors by country

Variable	IRR	95% CI (cluster-robust)	p-value
ONI (continuous)	0.851	0.359 - 2.017	0.714
Cyclone exposure	3.878	1.709 - 8.799	0.001
Flood exposure	1.928	0.558 - 6.661	0.299
Drought exposure	3.887	0.496 - 30.464	0.196

Under this conservative approach, cyclone exposure remained statistically significant (p = 0.001), whereas the intervals for flood and drought widened and included the null value. This is an expected consequence of the small number of clusters and supports a cautious, associational interpretation of the estimates.

Table S7. Sensitivity analysis: Combined climate-event & binary El Nino/La Nina indicators

Variable	IRR	95% CI	p-value
ONI (continuous)	0.912	0.789 - 1.054	0.214
Any climate event	3.201	2.648 - 3.868	<0.001

Binary El Nino/La Nina

Variable	IRR	95% CI	p-value
El Nino (ONI ≥ +0.5)	1.047	0.766 - 1.431	0.772
La Nina (ONI ≤ −0.5)	1.293	0.858 - 1.946	0.219
Cyclone exposure	3.990	2.647 - 6.013	<0.001
Flood exposure	2.035	1.544 - 2.683	<0.001
Drought exposure	3.866	2.908 - 5.141	<0.001

Model AIC = 11385.2. A single combined climate-event indicator was associated with a 3.2-fold higher weekly cholera incidence.
Model AIC = 11385.4. When continuous ONI was replaced by binary El Nino/La Nina indicators, neither ENSO phase was independently associated with incidence, while cyclone, flood and drought exposures remained robust (all p < 0.001). Across specifications the borderline inverse ONI association in the primary model was not robust, and is interpreted with caution.
