# Supplementary material for: Cholera trends in the WHO African region and temporal co-occurrence with El Niño, tropical cyclones, floods and droughts: 2022–2023
Source: BMJ Public Health. 2026 Aug 12;4(Suppl 1):e004479. doi: 10.1136/bmjph-2025-004479 (PMC13479493; doi:10.1136/bmjph-2025-004479)
Supplement: Supplementary file 2 [file bmjph-4-Suppl_1-s002.pdf]

Supplementary Figure S1. Weekly cholera epidemic curves in four highest-burden countries, 2022-2023

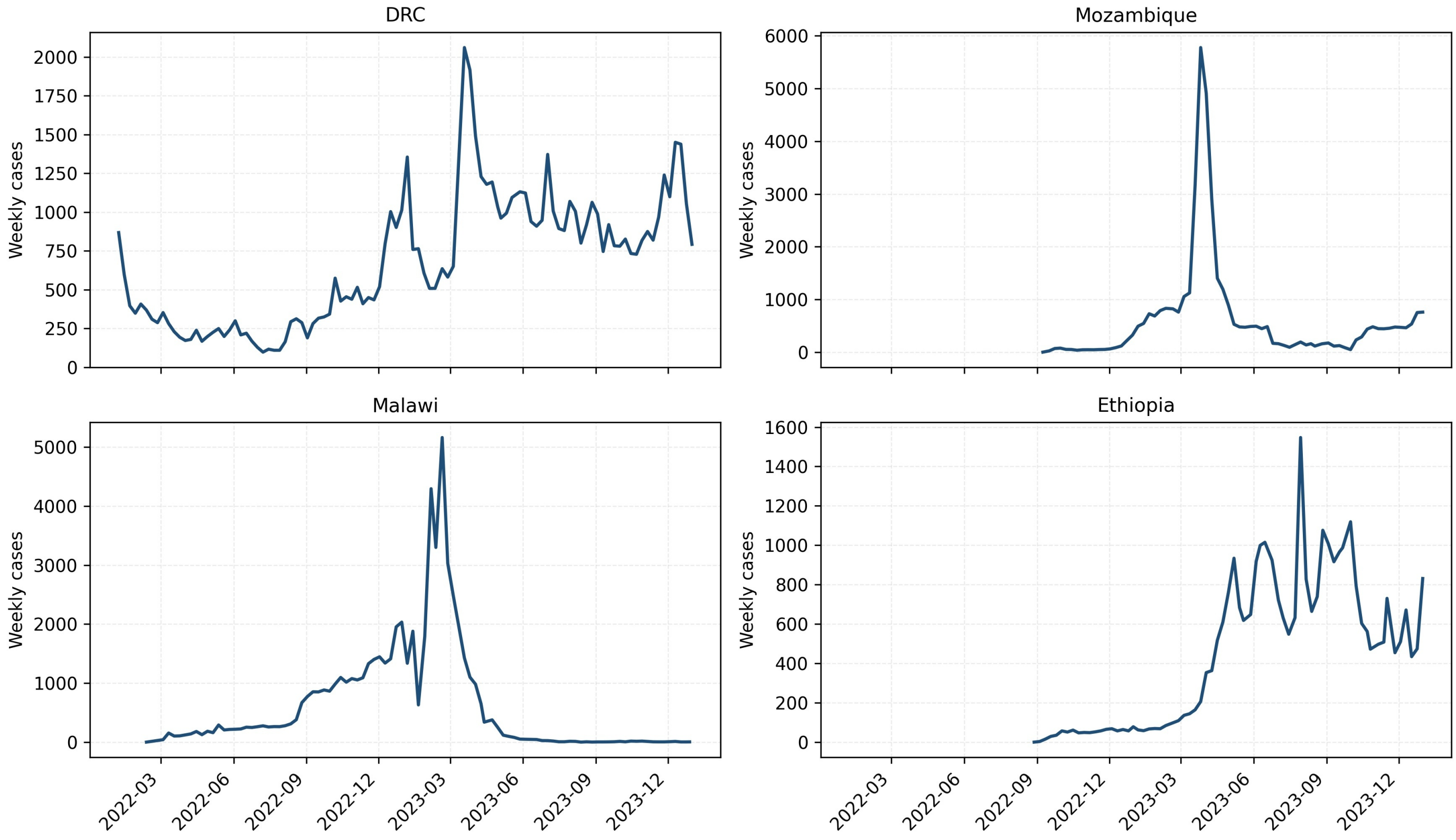

Data source: WHO AFRO weekly surveillance dataset used in the primary analysis.
