# Supplementary material for: Cholera trends in the WHO African region and temporal co-occurrence with El Niño, tropical cyclones, floods and droughts: 2022–2023
Source: BMJ Public Health. 2026 Aug 12;4(Suppl 1):e004479. doi: 10.1136/bmjph-2025-004479 (PMC13479493; doi:10.1136/bmjph-2025-004479)
Supplement: Supplementary file 3 [file bmjph-4-Suppl_1-s003.pdf]

Supplementary Figure S2. Trend of cholera cases across WHO AFRO subregions, January 2022 – December 2023

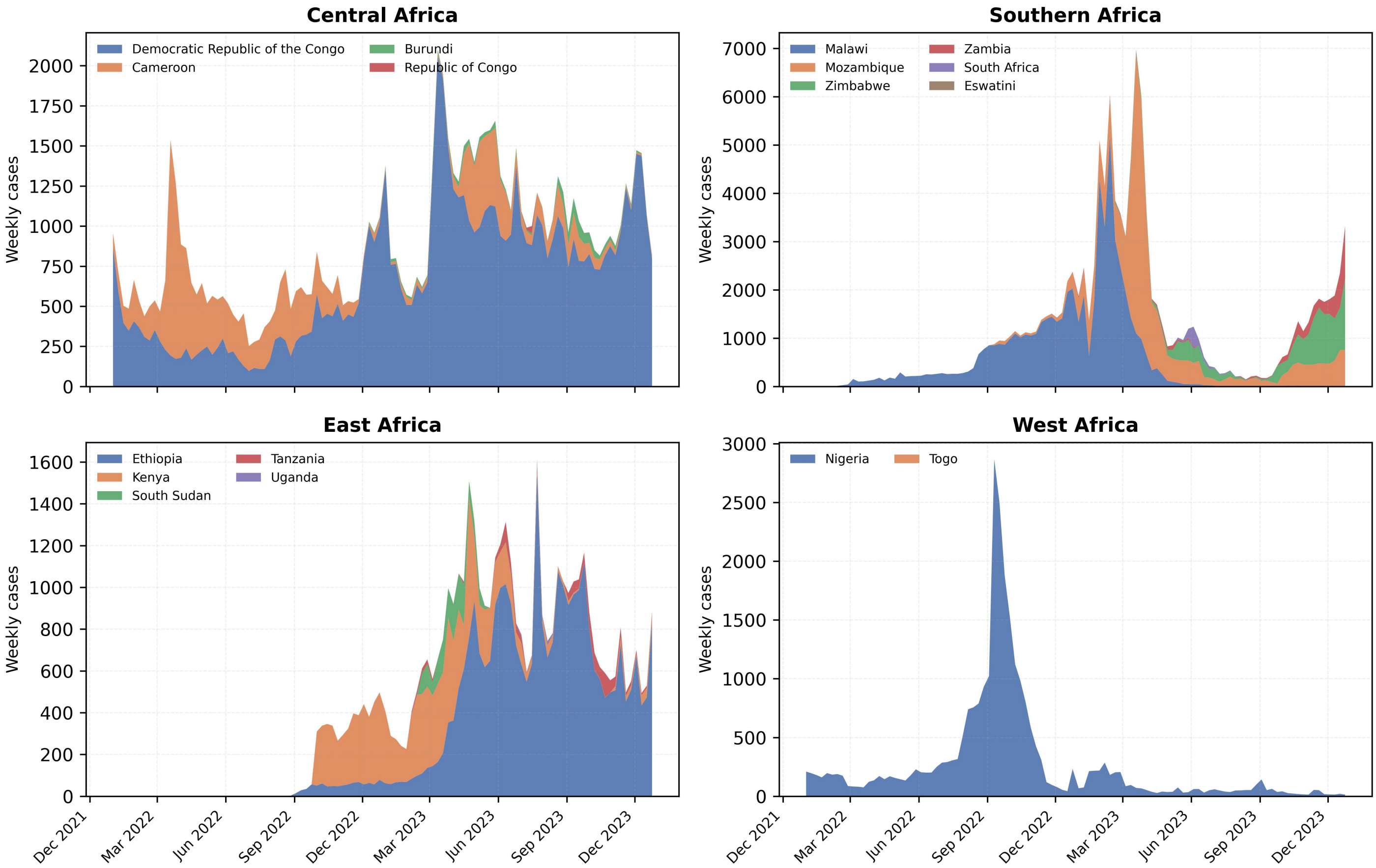

Panels show weekly reported cholera cases by country within each WHO AFRO subregion (raw counts, no smoothing). Moved from the main manuscript.
